# Multimodal use of indocyanine green endoscopy in neurosurgery: a single-center experience and review of the literature

**DOI:** 10.1007/s10143-017-0858-4

**Published:** 2017-05-06

**Authors:** Giuseppe Catapano, Francesco Sgulò, Lili Laleva, Laura Columbano, Iacopo Dallan, Matteo de Notaris

**Affiliations:** 1 Department of Neuroscience, Neurosurgery Operative Unit, “G. Rummo” Hospital, Via Pacevecchia no. 53, 82100 Benevento, Italy; 2 Division of Neurosurgery, Department of Neurological Sciences, Università degli Studi di Napoli Federico II, Naples, Italy; 3 Department of Neurosurgery, Tokuda Hospital Sofia, Sofia, Bulgaria; 4 First Otorhinolaryngologic Unit, Azienda Ospedaliero—Universitaria Pisana, Pisa, Italy

**Keywords:** Indocyanine green videoangiography, Endoscopy, Transsphenoidal, Third ventricle, Aneurysm, Brain tumors

## Abstract

During the last 10 years, microscope-integrated indocyanine green fluorescence (m-ICG) has been widely used for assessing real-time blood flow during aneurysm surgery. More recently, an endoscope-integrated indocyanine green fluorescence (e-ICG) has been adopted as a versatile tool during different endoscopic neurosurgical procedures. The purpose of the present report is to evaluate multimodal applications of e-ICG during different endonasal, intraventricular, aneurysm and brain tumor surgeries and provide technical nuances. In addition, we reviewed the literature and identified and compare several overlapping case series of patients treated via an endoscopic integrated indocyanine green fluorescence technique. A total of 40 patients were retrospectively evaluated. Patients were divided into four main groups: (1) endoscopic endonasal approaches (*n* = 14); (2) ventricular endoscopic approach including patients undergoing third ventriculostomy (*n* = 8) and tumor biopsy (*n* = 1); (3) aneurysms surgery (*n* = 9); and (4) brain parenchymal tumors (*n* = 8). All patients were successfully treated using the e-ICG dynamic endoscopic visualization, and there were no perioperative complications. Such unique features open up a promising field of applications beyond the use of m-ICG in different surgical field due to the longer duration of e-ICG fluorescence up to 35 ± 7 min. E-ICG represents a new and effective technique for longer real-time visualization of vascular structures preserving normal tissues and functions during different transcranial and endonasal approaches. As the technology and e-ICG resolution improves, the technique has the potential to become a critical tool for different applications in neurosurgery.

**Electronic supplementary material:**

The online version of this article (doi:10.1007/s10143-017-0858-4) contains supplementary material, which is available to authorized users.

## Introduction

Indocyanine green (ICG) angiography has become a well-established technology in several surgical fields, including ophthalmology, general, plastic, vascular, cardiac and head and neck surgery [1–8]. The principle of fluorescence imaging used during ICG videoangiography is simple: illuminate the tissue of interest with light at the excitation wavelength (about 750 to 800 nm) while observing it at longer emission wavelengths (over 800 nm). The confinement of indocyanine to the vascular compartment through binding with plasma proteins, the low toxicity and the rapid excretion, almost exclusively into the bile, represents the main advantages of this visualization tool.

Concerning neurosurgery, microscope-integrated indocyanine green (m-ICG) angiography was firstly adopted in vascular surgery by Raabe [9, 10] in 2003. The technique enabled the real-time evaluation of exposed vessel patency during surgery, configuring as an adequate alternative to intraoperative arteriography (IOA) and a clinical tool to reduce rates of incomplete clipping or occlusion of surrounding vessel aneurysms in vascular surgery. During the years that followed, increased versatility and minimized invasiveness relative to IOA has facilitated the use and diffusion of m-ICG beyond the field of vascular surgery, i.e., during brain and spinal procedures as well as pituitary surgery [11–15]. More recently, with the development of an endoscope with integrated green indocyanine (e-ICG) filter first adopted in vascular surgery to verify the patency of vessels hidden from microscopic view, the increased effectiveness of this novel tool in different fields of neurosurgery has also been reported. Currently, the four main application fields of e-ICG are neurovascular [16–18], endoscopic endonasal approaches [19–21], ventricular surgery [22, 23] and neurooncological [22, 24]. As a matter of fact, employing the e-ICG dynamic technique in different surgical scenarios, such as in fluorescence-guided resection of brain and pituitary tumors as well as neuroendoscopic approaches, the management of a wide spectrum of pathological conditions shows promise for future development.

We therefore designed a *multimodal* study to evaluate the safety and feasibility of intravenous application of indocyanine in combination with the e-ICG visualization in patients undergoing various endonasal, ventricular, aneurysm and brain tumor surgeries and perform a comprehensive review of the literature.

## Material and methods

The present retrospective study examined a total of 40 consecutive patients treated in our Institution from January 2015 to June 2016. All interventions were performed using the e-ICG as the sole visualizing tool with the exception of aneurysm and brain tumor surgery in which the endoscope was used in combination with the microscope (endoscope-assisted technique). In all cases, neuronavigation (Medtronic S7 StealthStation, Louisville, CO, USA) was used to confirm the intraoperative findings of e-ICG. The patients were divided into four major groups according to the surgical approach: *endonasal*, *ventricular*, *vascular* and *brain tumor* procedures*.* Two different endoscopes were used: a dedicated ICG-integrated endoscope 5.8 mm in diameter and 19 cm in length, (Karl Storz, Tuttlingen, Germany) coupled to an IMAGE1 S camera system (Karl Storz, Tuttlingen, Germany) and a Lotta ventriculoscope (Karl Storz, Tuttlingen, Germany) combined to an external optical filter allowing ICG fluorescence visualization.

A 0.2–0.5-mg/kg intravenous single bolus of ICG was injected by the anesthesiologist during surgery in all groups except for vascular and extended endoscopic endonasal groups in which we used a second injection. Near-infrared excitation of the ICG fluorescence (780–820 nm) was obtained using a D-light P (Karl Storz) and routine toggling between white light and ICG illumination modes was achieved via a foot switch. In addition to standard images, five visualization enhancement modes, supplied by the IMAGE1 S technology (Clara, Chroma, Clara + Chroma, SPECTRA A and B), were utilized and compared during surgery.

Concerning the patient population, the endonasal group included 14 cases: 6 pituitary adenomas (4 non secreting and 2 ACTH secreting macroadenomas), 3 tuberculum sellae meningiomas, 2 clival chordomas, 1 craniopharyngioma, 1 nasopharyngeal carcinoma, and 1 suprasellar epidermoid cyst. All patients underwent an endoscopic endonasal approach. The ventricular group consist of one intraventricular tumor and eight non-communicating hydrocephalus. In all cases, an endoscopic third ventriculostomy (ETV) was performed via a right-sided pre-coronal approach. The vascular group included nine middle cerebral artery aneurysms initially presented without subarachnoid hemorrhage. In one case, there were two simultaneous aneurysms successfully clipped during the same surgery. In all cases, the patients underwent microsurgical clipping via a frontolateral approach. Microscopic (OPMI Pentero operating microscope, Carl Zeiss, Oberkochen, Germany) and endoscopic indocyanine green videoangiographies were contemporary performed to compare pre- and post-clipping images. Brain tumor group included eight intracranial tumors; each patient underwent a microsurgical endoscope assisted resection via different transcranial approaches: two meningiomas (spheno-orbital and frontal convexity), one third ventricle, two frontal and two temporal lobe gliomas, and one parietal lobe metastasis of ovarian carcinoma.

### Literature review

PubMed and Medline databases were searched with combinations of the search terms: “endoscopic indocyanine and third ventriculostomy”, “endoscopic indocyanine and transsphenoidal surgery”, “endoscopic indocyanine and skull base surgery”, “endoscopic indocyanine and aneurysm”, “endoscopic indocyanine and brain tumors” and “endoscopic indocyanine in neurosurgery” References contained within these papers were reviewed, including case reports. Overall, 95 studies were identified. Non-English articles, pre-clinical papers, studies including patients treated using microscopic-ICG technology alone were excluded from our analysis. This resulted in a total of 10 papers which were included in our analysis [16, 17, 19–23, 25–27].

## Results

### Case series

Endoscope-integrated indocyanine green fluorescence procedures were successfully performed in all patients. There were no intraoperative surgical complications reported. Post-operative complication occurred in two cases in the endonasal group and they were represented by transient diabetes insipidus. Adverse events due to the use of the dedicated ICG endoscope and/or ICG administration did not occur. ICG fluorescence was visualized on the video screen approximately 10–15 s after its intravenous injection. Interestingly, a prolonged fluorescence visualization time (the mean of 35 ± 7 min) was possible using the ICG endoscope compared to the operative microscope (15 s) [17]. Specific use and tips related to the surgical approach, the pathology and the visualization enhancement were different in relation to each procedure (Table 1).

**Table 1:** Specific ICG use and tips related to the surgical approach, the pathology, and e-ICG visualization enhancement in relation to each surgical procedure

Type of approach	e-ICG use and tips
Endonasal	Standard sellar approach: suggested use 1 shot 25 mg - Significantly enhanced the localization of parasellar segments of both internal carotid arteries (ICAs) and superior and inferior intercavernous sinuses before dura opening - Pituitary gland remained fluorescent until the end of the procedure so to easily preserve it during tumor excision
	Extended approach: suggested use 2 shots 12.5 mg It was possible to identify and divide the vascular structures into the following: *submucosal* (sphenopalatine artery and its posterior septal branch: very useful to harvest the nasoseptal flap), *underneath the bone* (clinoidal and petrosal segment of the internal carotid arteries), *epidural* (dural arteries, superior and inferior intercavernous sinuses), *intradural* (optic chiasm perforators, superior hypophyseal arteries, anterior communicating artery, anterior cerebral artery, pituitary gland and stalk)
Ventricular	Suggested use: 1 shot 25 mg - Clearly showed the course of the basilar and posterior cerebral arteries, and it was very useful to determine the safest site for ventriculostomy - Concerning the third ventricle tumor, ICG administration enhanced visualization of tumor margins and was useful to identify proper sites to biopsy
Vascular	Suggested use: 2 shots 12.5 mg - Aneurysm morphology, perforating vessels preservation - Excellent visualization window for fluorescence angiography during the pre- and post-clipping steps of surgery
Brain tumors	Suggested use: 1 shot 25 mg Intraaxial: Identification of the tumor based on the differences in fluorescence intensity before surgical removal due to blood-brain barrier disruption Extraaxial: Useful information on the tumoral and peritumoral vessels. Post-resection, the patency of the peritumoral vessels could be assessed

#### Endonasal group

**Fig. 1:**
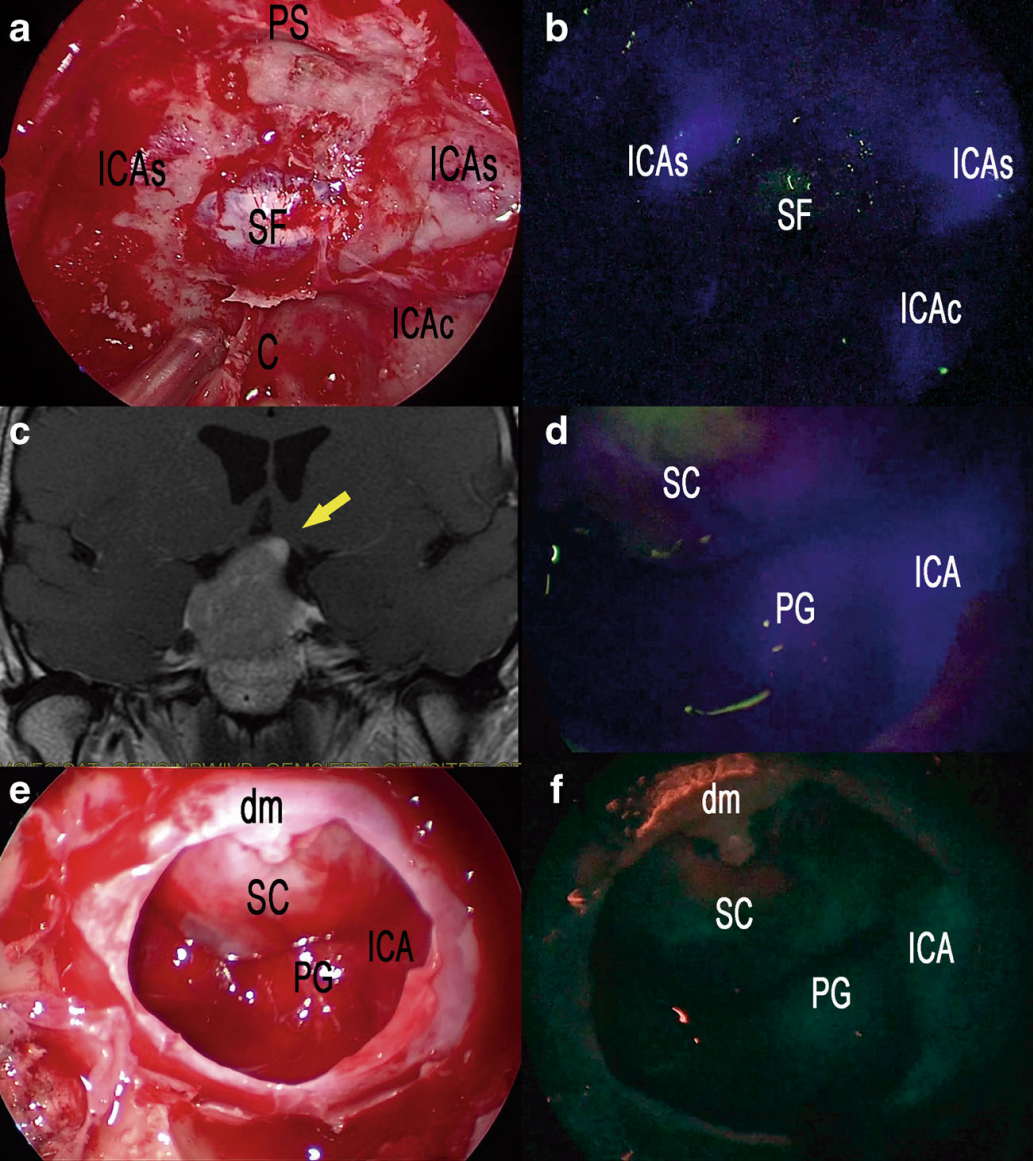
Standard endoscopic endonasal approach to the sella. **a** The photo demonstrates the surgical field during sphenoidal step of the approach and the appearance under white light of the posterior wall of the sphenoid sinus. **b** The same surgical field as in **a** under near-infrared light after injection of ICG. **c** Post-contrast T1-weighted brain magnetic resonance imaging (MRI), coronal view, showing the pituitary macroadenoma with elevation and compression of the optic chiasm. The *yellow arrow* shows the position of the pituitary gland. **d** ICG fluorescence highlighting vascular structures within the intradural step of the approach. **e** The photo demonstrates the same field of view and the appearance of sellar area under white light. **f** The same surgical field under near-infrared light after injection of ICG using SPECTRA-A mode. *SC* suprasellar cistern, *PG* pituitary gland, *SF* sellar floor, *C* clivus, *ICA* internal carotid artery, *ICAs* parasellar segment of the internal carotid artery, *ICAc* clival segment of the internal carotid artery, *dm* dura mater, *PS* planum sphenoidale

**Fig. 2:**
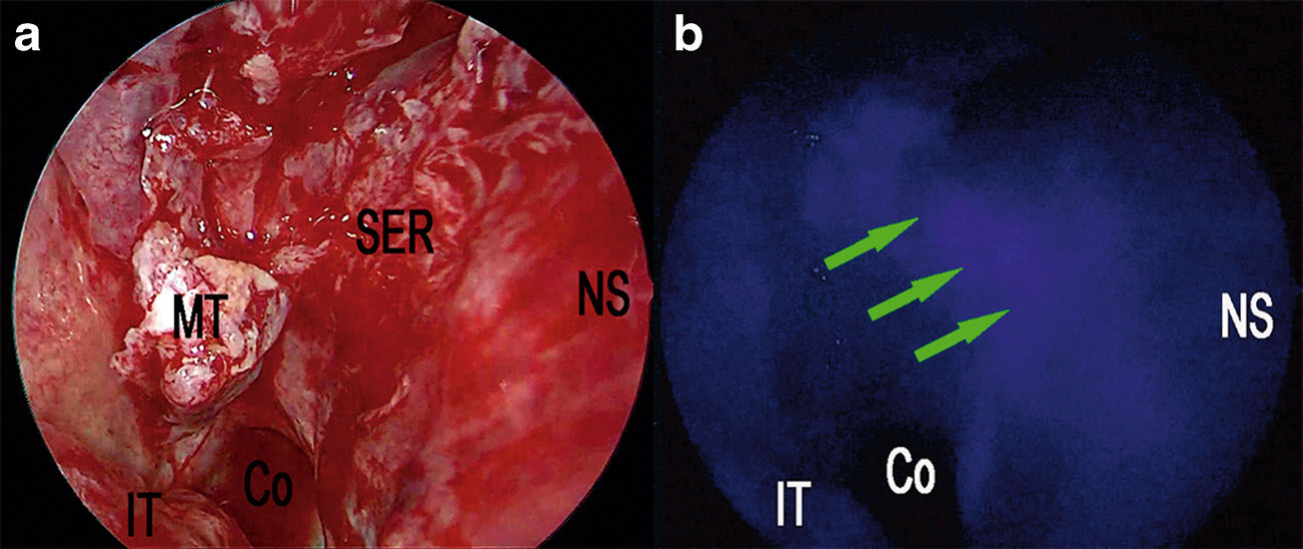
Nasal step of extended endoscopic endonasal approach (**a**). Right nostril, harvesting of a pedicled nasal septal flap based on the posterior nasal septal artery (**b**). The same surgical field under near-infrared light after injection of ICG fluorescence highlighting vascular structures within nasal septum; the *green arrows* show the submucosal position of the posterior nasal septal artery. *Co* choana, *IT* inferior turbinate, *MT* middle turbinate, *SER* sphenoethmoid recess, *NS* nasal septum

**Fig. 3:**
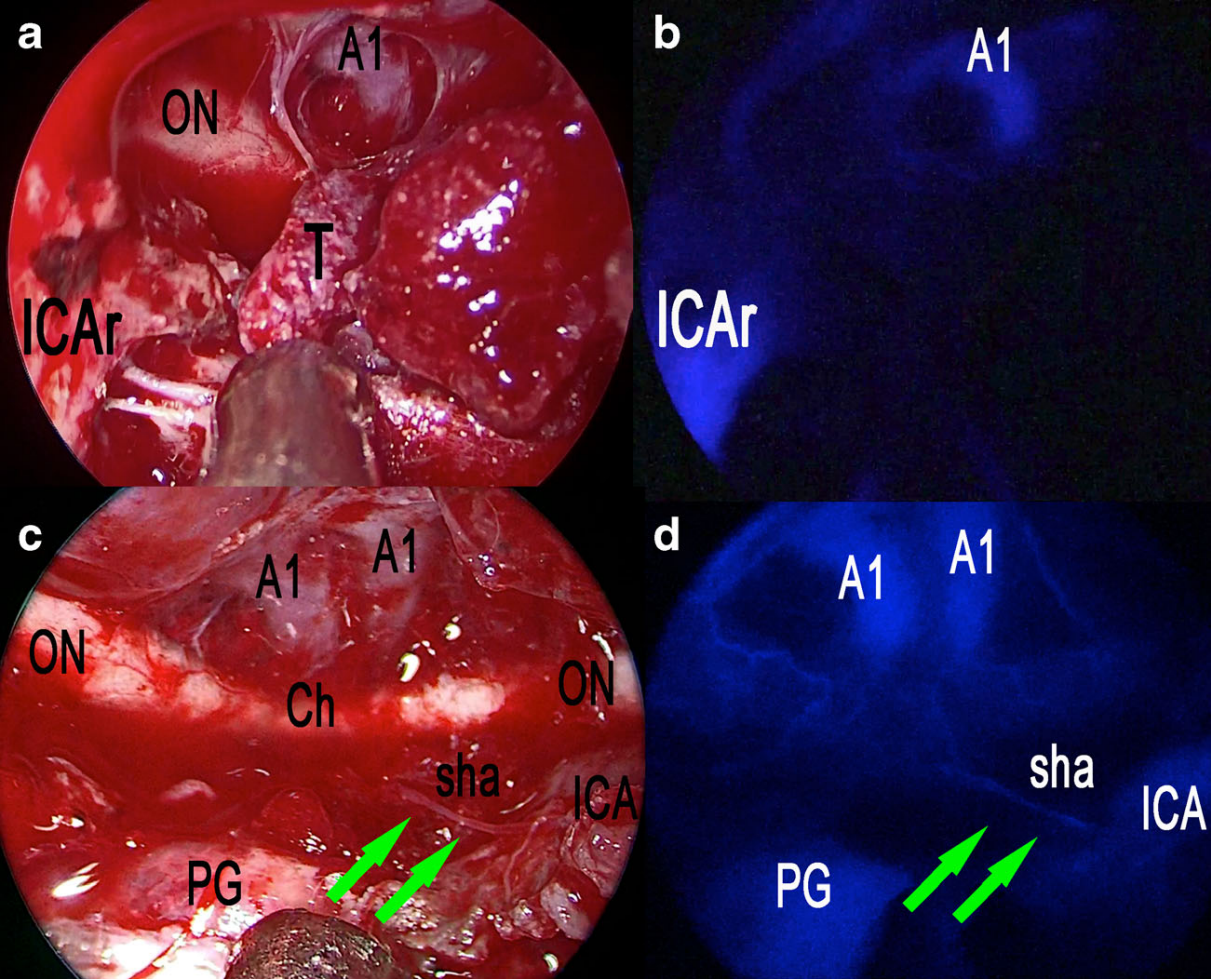
Extended endoscopic endonasal transtuberculum/transplanum approach, removal of a tuberculum sellae meningioma. **a** The photo demonstrates the surgical field during tumor removal step and the appearance under white light. **b** The same surgical field as in **a** under near-infrared light after injection of ICG. **c** The photo demonstrates the surgical field after the complete excision of the tumor and the appearance under white light. **d** The same surgical field as in **c** under near-infrared light after injection of ICG. *ON* optic nerve, *A1* anterior cerebral artery, *T* tumor, *ICAr* right internal carotid artery, *Ch* optic chiasm, *PG* pituitary gland, *sha* superior hypophyseal artery; *green arrows* show the course of the superior hypophyseal artery

**Fig. 4:**
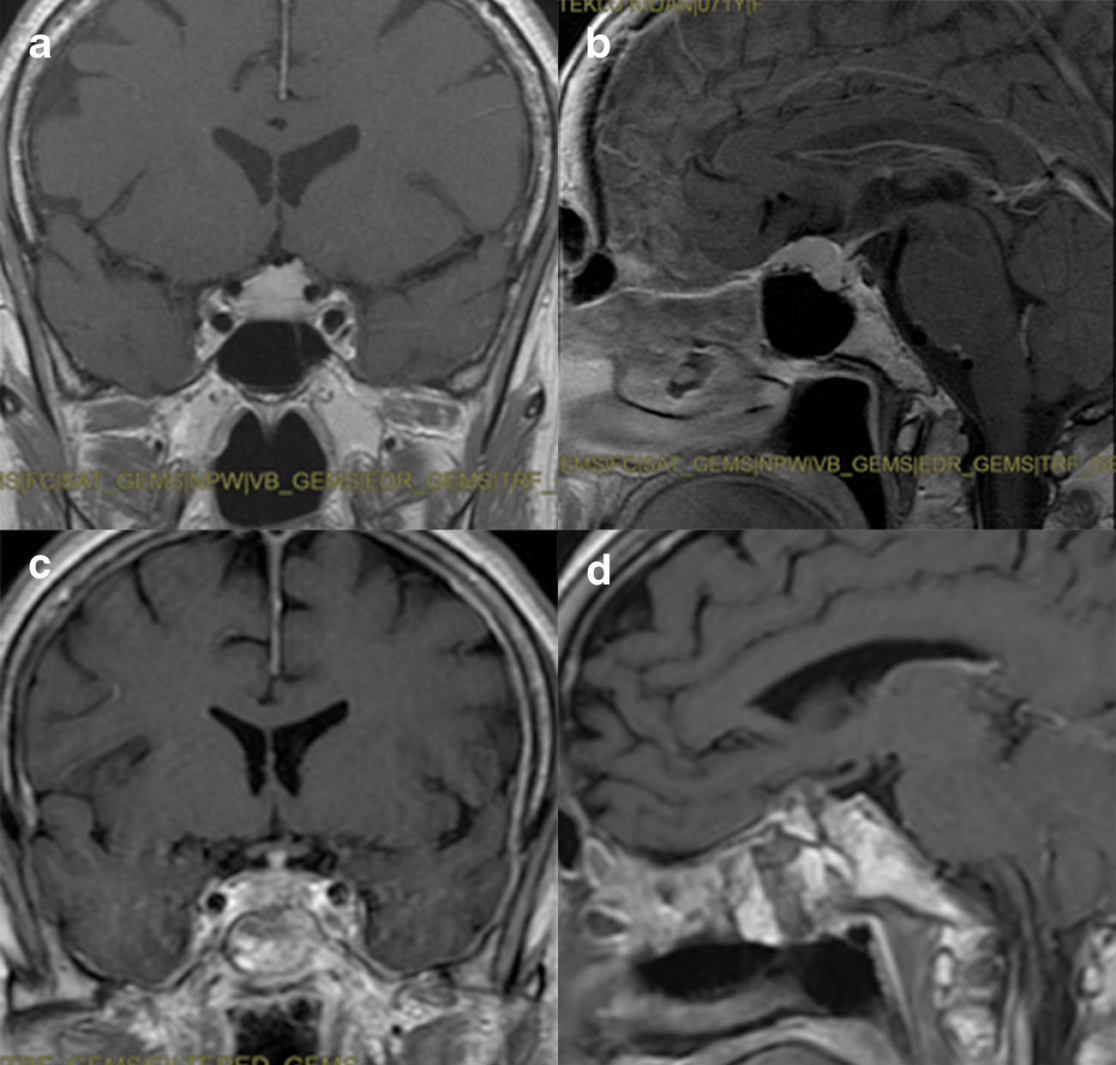
Post-contrast T1-weighted brain MRI, coronal and sagittal view, showing preoperative (**a**, **b**) and postoperative (**c**, **d**) images of a tuberculum sellae meningioma

**Fig. 5:**
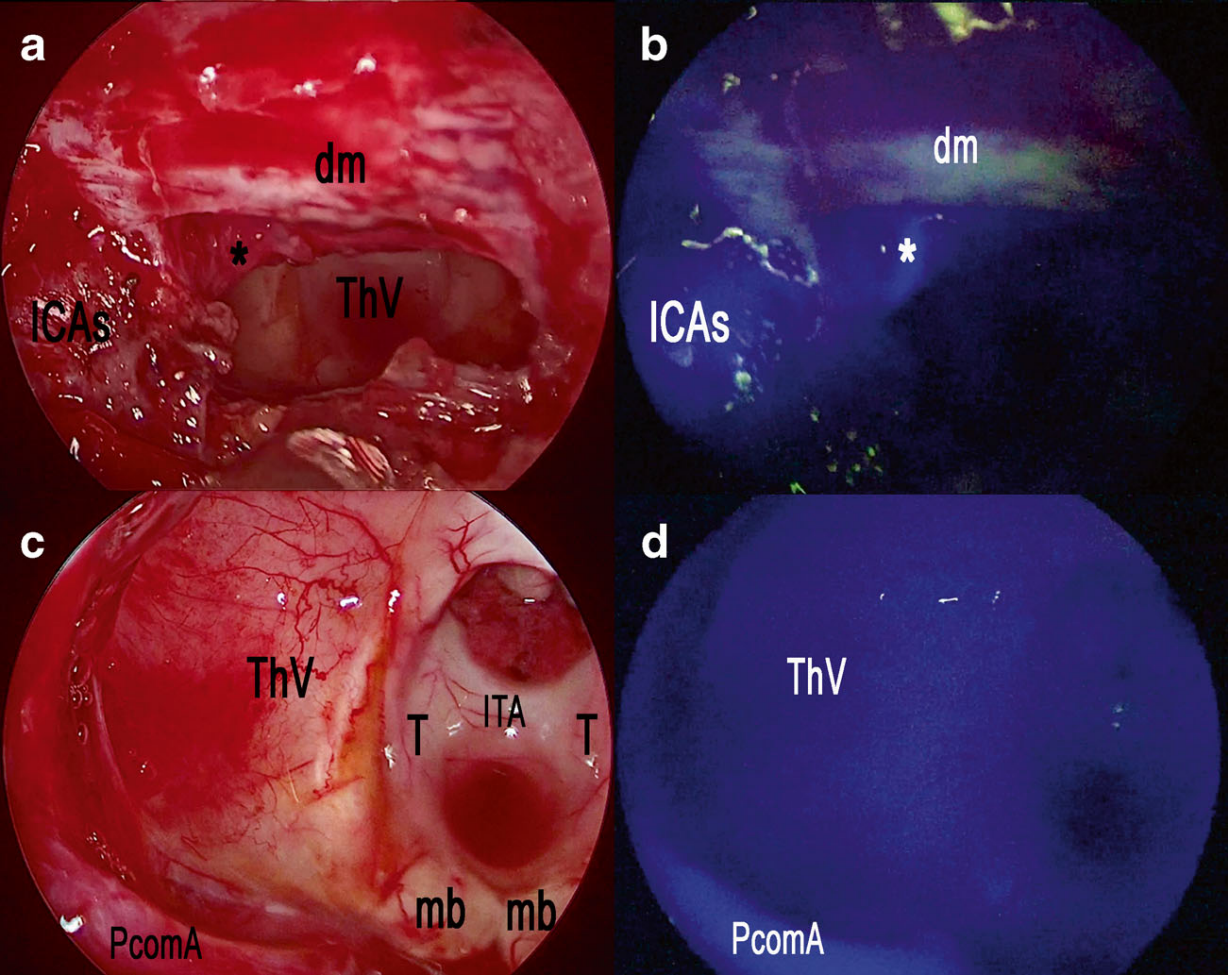
Extended endoscopic endonasal transtuberculum/transplanum approach, removal of a suprasellar craniopharyngioma. **a** The photo demonstrates the surgical field during the removal of the tumor and the appearance under white light. **b** The same surgical field as in **c** under near-infrared light after injection of ICG. **c** The photo demonstrates the surgical field after the complete removal of the tumor and the appearance under white light. **d** The same surgical field as in **c** under near-infrared light after injection of ICG. *ICAs* parasellar segment of the internal carotid artery, *asterisk* superior hypophyseal artery, *dm* dura mater, *ThV* third ventricle, *T* thalamus, *ITA* interthalamic adhesion, *PcomA* posterior communicating artery, *mb* mammillary body

**Fig. 6:**
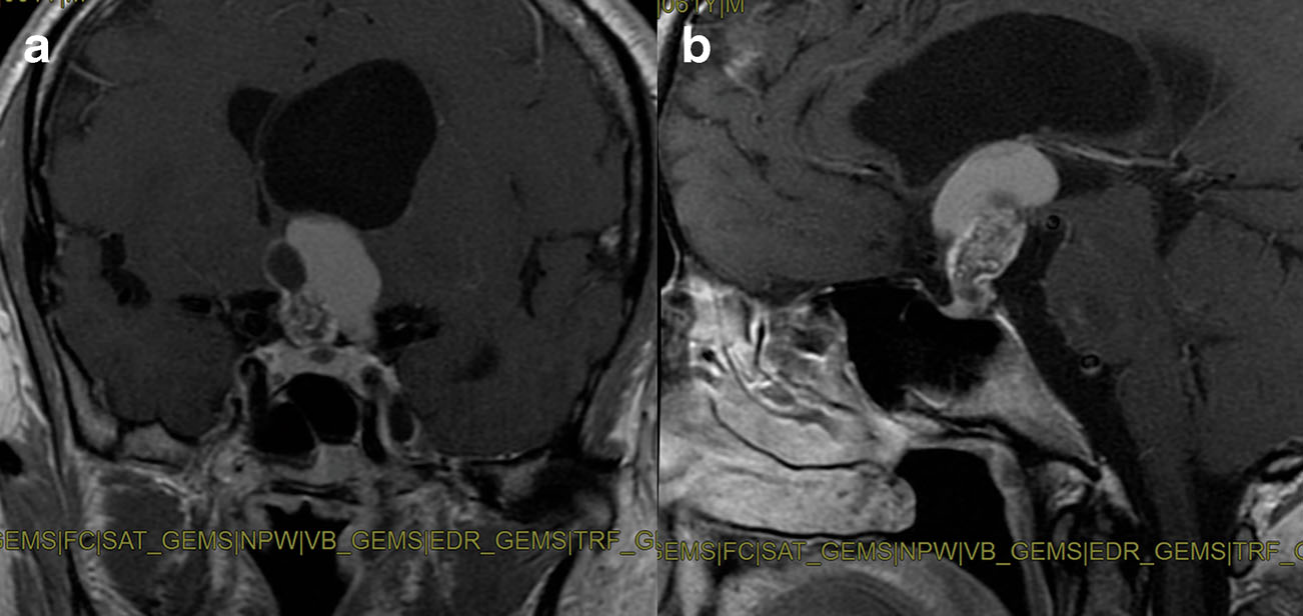
Post-contrast T1-weighted brain MRI, coronal (**a**) and sagittal (**b**) views, showing a suprasellar craniopharyngioma with a large third ventricular extension

Concerning cases with a standard sellar approach, a single 25 mg shot of ICG was injected after the anterior sphenoidotomy: e-ICG significantly enhanced the localization of parasellar segments of both internal carotid arteries (ICAs) (Fig. 1a, b) and superior and inferior intercavernous sinuses before dura opening. Interestingly, during pituitary adenomas removal, the gland remained fluorescent until the end of the procedure so to easily preserve it during tumor excision. SPECTRA-A, among the IMAGE1 S visualization enhancement modes, appeared particularly useful in pituitary gland detection to perform a safe and radical tumor resection (Fig. 1c–f; Video 1). Operating continuously under ICG-mode was also found to be beneficial to localize the bleeding sites under ICG illumination. In the case of *extended* endoscopic endonasal approach, two shots of 12.5 mg ICG were administered: the first was injected during the nasal step to precisely tailor the nasoseptal flap thus localizing the sphenopalatine artery and its septal branch (Fig. 2). The same doses also allowed subsequential localization of the internal carotid arteries and epidural anatomical structures (dural arteries, superior and inferior intercavernous sinus). A second dose of 12.5 mg of ICG was then injected during tumor removal to precisely localize and check the patency of the neurovascular structures as shown in Fig. 3, during an extended endoscopic endonasal transtuberculum/transplanum approach to remove a tuberculum sellae meningioma (Fig. 4). The same technique was also performed to remove a suprasellar craniopharyngioma (Fig. 5) with a large intraventricular extension (Fig. 6). This way it was possible to identify and divide the recognized vascular structures into the following: *submucosal* (sphenopalatine artery and its septal branch, very useful to harvest the nasoseptal flap), *underneath the bone* (parasellar and paraclival segments of the internal carotid arteries), *epidural* (dural arteries, superior and inferior intercavernous sinuses) and *intradural* (optic chiasm perforators, superior hypophyseal arteries, anterior communicating artery, anterior cerebral artery, pituitary gland and stalk). Fluorescence confirmed the vascular anatomical information acquired through the neuronavigation system, specially the position of the pituitary gland during all procedures (Fig. 2; Video 1). However, in this group, it was mandatory to ensure a clear and bloodless operative field before using ICG to avoid “diffuse” and “false positive” fluorescence visualization.

#### Ventricular group

**Fig. 7:**
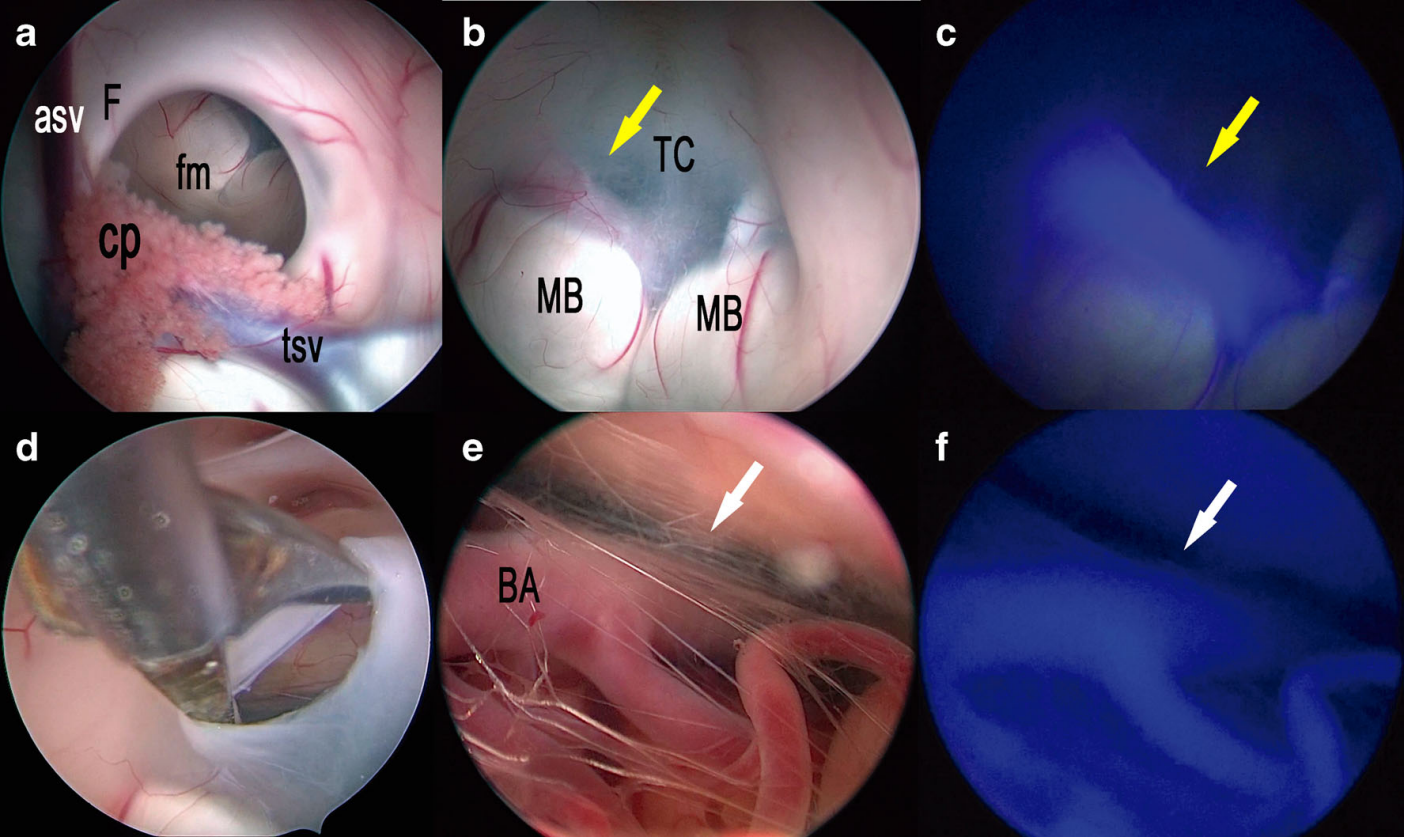
Right frontal endoscopic third ventriculostomy. **a** The photo demonstrates the surgical field in the lateral (**a**) and third (**b**) ventricles under white light. **c** The same surgical field as in **b** under near-infrared light after injection of ICG. **d** The photo demonstrates the surgical field during the opening of the third ventricle floor in the area of the tuber cinereum and the endoscopic exploration of the interpeduncular cistern (**e**) under white light. **f** The same surgical field as in **e** under near-infrared light after injection of ICG. *asf* anterior septal vein, *cp* choroid plexus, *fm* foramen of Monro, *F* fornix, *tsv* thalamostriate vein, *MB* mammillary body, *BA* basilar artery; *yellow arrow* shows the course of the posterior cerebral artery; *white arrow* shows the course of the basilar artery

In all cases, a single 25 mg bolus of ICG was injected when the third ventricle floor was under vision, prior to ventriculostomy. While performing such procedure, fenestration of the third ventricle floor should be performed between the mammillary bodies and the infundibular recess, at the most transparent site (*tuber cinereum*). Under white light visualization, the localization of the typically invisible basilar artery is critical to avoid vessel injury during the procedure and the fenestration should be made anterior to the artery complex. In all cases, a ventriculoscope coupled with a dedicated external optical filter allowing transmission of ICG fluorescence clearly showed the course of the basilar and posterior cerebral arteries and it was very useful to determine the site for ventriculostomy ensuring total safety (Fig. 7; Video 1). Concerning the case of the third ventricle tumor, histology showed a pilocytic astrocytoma and ICG administration enhanced visualization of tumor margins and was useful to identify proper sites to biopsy as already demonstrated by Tsuzuki et al. [22].

#### Vascular group

**Fig. 8:**
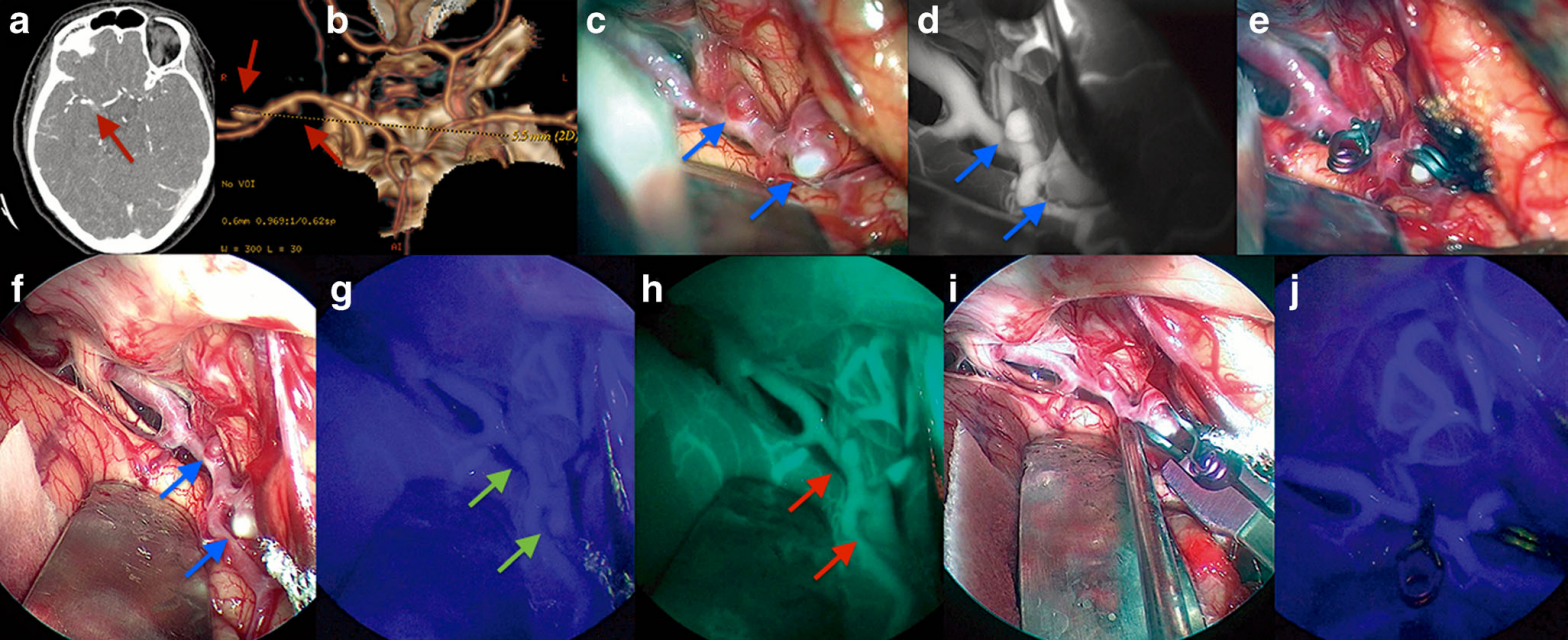
Intraoperative findings during two middle cerebral artery aneurysms clipping cases via a right frontolateral approach. High-resolution computer tomography (CT) angiography and CT-based 3D reconstruction of two middle cerebral artery aneurysms (**a**, **b**); *red arrows* show the position of the aneurysms. **c** Right Sylvian fissure opening under microscopic view and white light; *blue arrows* show the position of the aneurysms. **d** The same surgical field as in **c** under indocyanine microscopic integrated view; *blue arrows* show the position of the aneurysms. **e** Clipping of the two middle cerebral aneurysms under microscopic view and white light. **f** Same case, right Sylvian fissure opening under endoscopic assisted view and white light; *blue arrows* show the position of the aneurysms. **g** The same surgical field as in **f** under near-infrared light after injection of ICG; *green arrows* show the position of the aneurysms. **h** The same surgical field as in **g** under near-infrared light after injection of ICG using SPECTRA-A mode; *red arrows* show the position of the aneurysms. **i** Clipping of the two middle cerebral aneurysms under endoscopic assisted view and white light. **j** Post-clipping step of the two middle cerebral aneurysms under endoscopic assisted view and near-infrared light after injection of ICG

In such group, two doses of ICG were administered: one dose of 12.5 mg of ICG before aneurysm clipping and the second to confirm the correct position of the clip and the complete exclusion of the aneurysm from the parental artery. Using this technique, e-ICG provided routine guidance for complete clipping while demonstrating the patency of the parent and surrounding perforating arteries before and after clip placement in all cases (Fig. 8; Video 1). In aneurysm surgery, the fact that e-ICG fluorescence can be prolonged and visualized up to 42 min provided an excellent visualization window for fluorescence angiography during the pre-clipping step of surgery without the need of further ICG administration (Table 2). Moreover, the endoscopic technique enabled dynamic optimization of the field of view; this feature allowed for a more complete evaluation of the parent artery-aneurysm complex and the perforating arteries behind the aneurysm before and after clip placement in selected cases. Despite the long-lasting fluorescence during the e-ICG intervention, a second drug administration was mandatory for post-clipping evaluation because of “trapped” ICG filling into the aneurysm wall that could simulate an incomplete clipping.

**Table 2:** General aspects: pros and cons of e-ICG use

Pros	Cons
FDA approval: low toxicity and rapid elimination through bile excretion	Dedicated endoscope with a larger diameter (5.8 mm)
Fluorescence visualization window can be seen up to 42 min, much more than with the operative microscope.	External ICG filter does not allow the same image quality observed in the other procedures with a dedicated integrated ICG endoscope.
Versatility of such tool in different neurosurgical scenarios and complex vascular environments	Unavailability of angled telescopes limits direct operative view of blind corners.
Penetrate tissue up to 3/10 mm below the surface raises the possibility of identifying deeper lying vascular structures masked under various tissues	Very clear surgical field is mandatory in order to expose the desired vascular structure without the risk of false visual perception.
There is still room for future technical developments.	The lack of simultaneous visualization of fluorescent and non-fluorescent images in parallel

#### Brain tumor group

**Fig. 9:**
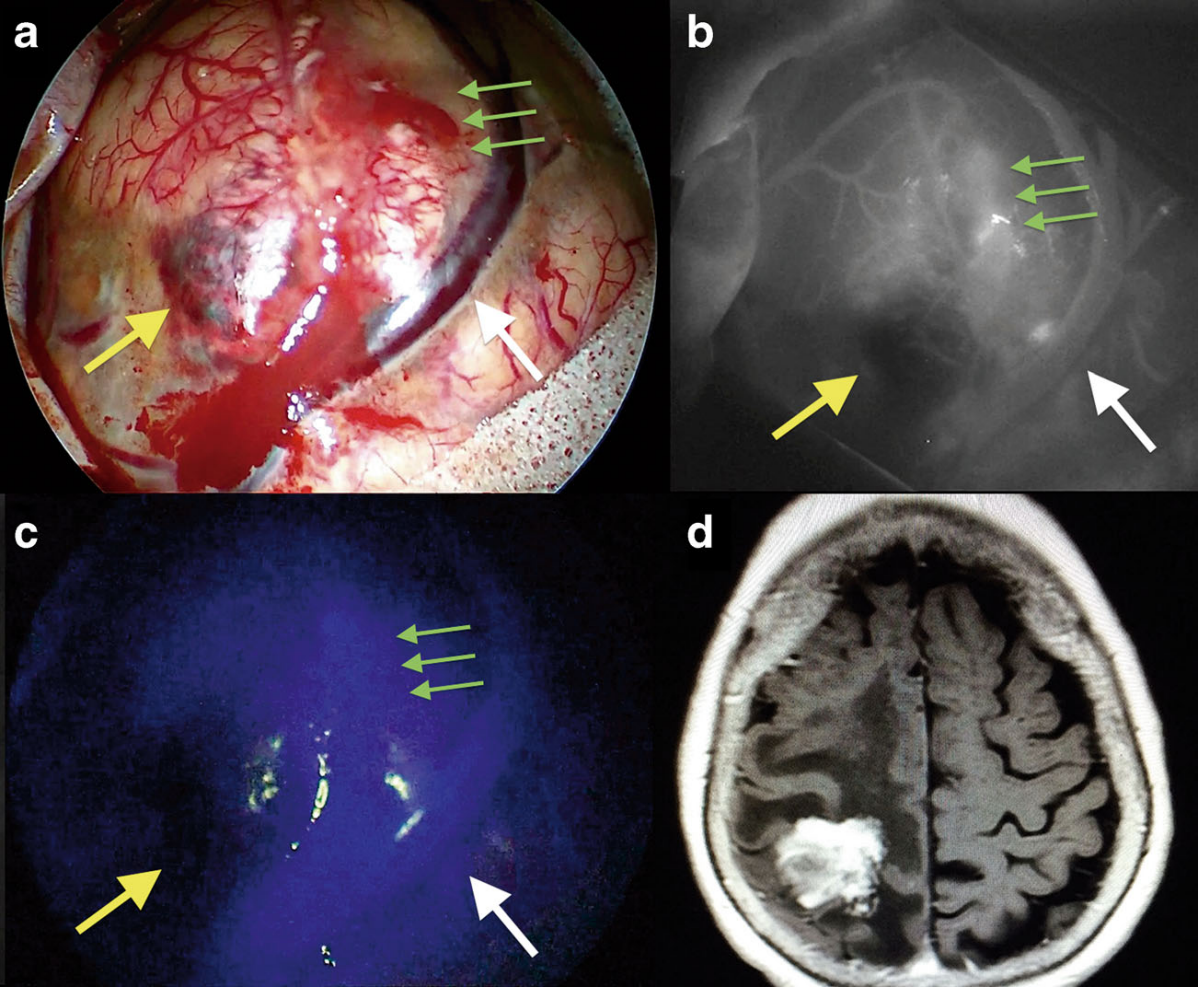
The intraoperative findings during a parietal lobe metastasis of ovarian carcinoma removal via a right parietal approach. **a** Microscopic view before tumor removal under white light; *yellow arrow* shows the necrotic part of the lesion while the *green arrows* show the blood-brain barrier disruption. **b** The same surgical field as in **a** under indocyanine microscopic integrated view; *yellow arrow* shows the necrotic part of the lesion while the *green arrows* show the blood-brain barrier disruption. **c** The same surgical field as in **b** under endoscopic assisted view and near-infrared light after injection of ICG; *yellow arrow* shows the necrotic part of the lesion while the *green arrows* show the blood-brain barrier disruption

**Fig. 10:**
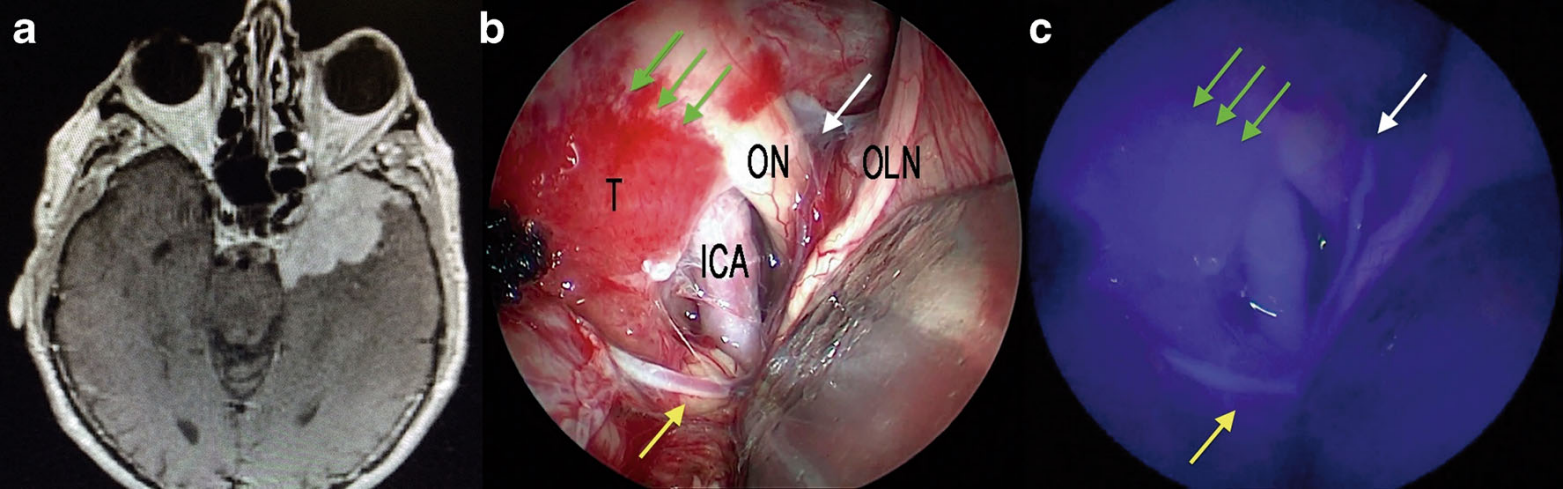
Intraoperative findings during spheno-orbital meningioma removal via a left pterional approach. Post-contrast T1-weighted brain MRI, axial view, showing the spheno-orbital meningioma extension. **b** Endoscopic assisted view showing the subfrontal exposure if the tumor under white light (**a**) and near-infrared light (**c**) after injection of ICG; *yellow arrow* shows a branch of the middle cerebral artery while the *green arrows* show the dural attachment of the lesion; the *white arrow* shows the a perforating artery over the left optic nerve

In all cases, a single 25 mg bolus of ICG was injected after opening of the dura and before tumor removal to assess tumor margins due to blood-brain barrier disruption and to visualize tumor and peritumor vascularization. Figure 9 presents the intraoperative findings during a parietal lobe metastasis of ovarian carcinoma removal via a right parietal approach. Employing the e-ICG approach in intrinsic brain tumor cases located within the cortical surface enabled the identification of the tumor based on the differences in fluorescence intensity before surgical removal due to blood-brain barrier disruption. Figure 10 shows the intraoperative findings during spheno-orbital meningioma removal via a left pterional approach. During extraaxial tumor resection, e-ICG provided useful information on the tumoral and peritumoral vessels. Post resection, the patency of the peritumoral vessels could be assessed and was especially useful for the veins.

### Literature review

Among 10 papers considered, 4 were included within the endonasal field, 2 within the ventricular field, 3 within the vascular field and 1 within the brain tumor field. All included studies are summarized in Table 3.

**Table 3:** Review of the literature focusing on the recent case series of patients treated via an endoscopic integrated indocyanine green fluorescence technique

Author, year	Number of patients	ICG endoscope	ICG dose and timing	Applications and advantages
				Endonasal
• Litvack ZN, 2012	15	Custom telescope with a selectable 790 nm filter	12.5–25 mg (bolus) × 2 or 3, after standard sellar approach, eventually during tumor exposure and a final injection after tumor removal	• Endoscopic transsphenoidal surgery for benign pituitary lesions • To distinguish pituitary tumors from normal tissue and to visually identify areas of dural invasion
• Hide T, 2015	38	0° telescope (5.8 mm/19 cm)	12.5 mg (bolus), after sphenoid sinus opening	• Standard and extended endoscopic transsphenoidal surgery • To confirm anatomical landmarks in real-time and to preserve blood supply of the optic nerves and pituitary
• Inoue A, 2015	10	0° telescope (5.8 mm/19 cm)	12.5 mg (bolus), on demand	• Endoscopic transsphenoidal surgery for sellar or intrasphenoidal sinus tumors • To display in real-time the anatomical relationships between the tumor and surrounding structures
• Simal Juliàn JA, 2016	2	Adapted 0° optical module	n/a	• Skull base expanded endonasal surgery • To localize ICA before sellar drilling and during the intradural exploration
				Ventricular
• Wachter D, 2013	11	Prototype of 0° rigid endoscope (5.8 mm in diameter)	0.2–0.5 mg/kg, after exposure of 3rd ventricle floor	• Endoscopic third ventriculostomy • To visualize the basilar artery and its perforators to reduce the risk of vascular injury
• Tsuzuki S, 2013	3	0° telescope (5.8 mm/19 cm)	12.5 mg (bolus) × 2 7.5 + 12.5 mg	• Endoscopic biopsy of intraventricular tumors • To consider the most appropriate region for biopsy
				Vascular
• Bruneau M, 2012	1	0° (5.8 mm/19 cm) NIR-optimized endoscope	20 mg (bolus) × 4, after aneurysm dissection and after clipping	• Clipping of unruptured anterior communicating artery aneurysm • To obtain additional information regarding aneurysm occlusion and patency of parent and branching vessels and small perforating arteries
• Mielke D, 2014	26	Prototype of 0° rigid endoscope (5.8 mm in diameter)	0.2–0.5 mg/kg, before and after clip placement	• Clipping of unruptured and ruptured cerebral aneurysms and comparison with m-ICG • To visualize hidden regions of the microsurgical field thanks to longer fluorescence, different and enlarged viewing angles compared with m-ICG
• Yoshioka H, 2015	9	30°/70° endoscopes (4 mm) with cut filter	n/a, before and after clip placement	• Clipping of cerebral aneurysms • To see in the dead angles of microscopic fluorescence video angiography for preventing unexpected occlusion of arteries around aneurysms
				Skull base tumors
• Yokoyama J, 2016	7	0° endoscope (5.8 mm/19 cm)	5 mg, after identification of tumor feeding artery	• Recurrent skull base cancer • To evaluate the blood supply to the tumor and to administrate superselective intraarterial chemotherapy

*n/a* not available

Concerning the endonasal field, the first experience was reported in 2012 by Litvack et al. [20] in a prospective study. The paper analyzes 16 patients (but 1 patient was excluded following discovery of a dye cross-allergy) undergoing endoscopic endonasal surgery for benign pituitary lesions using a standard endoscope with a near-infrared light source and an excitation wavelength filter. A dose of 12.5–25 mg was injected in bolus two or three times: first during the nasal step, a second injection was sometimes used during tumor exposure, and a final injection was performed at the end of the resection. In all considered cases, e-ICG successfully allowed to visually distinguish pituitary tumors (less fluorescent) from normal tissue and to identify dural invasion without reported complications.

The largest case series in the endoscopic endonasal field was reported by Hide et al. in 2015 [19]. They studied 38 patients with pituitary adenomas (*n* = 26), tuberculum sellae meningiomas (*n* = 4), craniopharyngiomas (*n* = 3), chordomas (*n* = 2), Rathke’s cleft cyst (*n* = 1), dermoid cyst (*n* = 1) and fibrous dysplasia (*n* = 1). A dedicated ICG endoscope (0°, 5.8 mm in diameter, and 19 cm in length) was used during a single dose of 12.5 mg, injected in bolus after sphenoid sinus opening. E-ICG assisted to confirm anatomical landmarks in real time and to preserve blood supply of the optic nerves and pituitary. The internal carotid artery (ICA) was identified through the dura mater and within the sphenoid sinus, if covered by thin bone.

Inoue et al. reported in 2015 [25] their experience concerning a multimodal assistant systems that generates a fusion model of preoperative three-dimensional (3D) computed tomography and magnetic resonance imaging (MRI) along with endoscopy with ICG fluorescence. They studied 10 patients who underwent endonasal surgery for sellar or sphenoidal sinus tumors. The authors used a 0° dedicated telescope (5.8 mm in diameter, 19 cm in length) and injected a single bolus of 12.5 mg of ICG. They reported that e-ICG resulted to be superior to the neuronavigation system to identify the internal carotid artery before opening the sellar floor.

Finally, Simal Juliàn et al. [21] reported in 2016 their experience, demonstrating that e-ICG provides a superior ability to detect the margins of the ICA compared with the Doppler technique and also provides enhancement of the artery through the bone of the skull base without the need for drilling. They reported two illustrative cases using an adapted 0° optical module; ICG dose and injection timing were not reported. The authors underlined two aspects: exposure of parasellar segments and paraclival segments of ICAs (intense fluorescent enhancement) before skull base drilling and then during the intradural step in which tumor resection, especially very closely to the ICA, was assisted by fluorescence under ICG-mode. The main limitations indicated by the authors were availability of only 0° optic lenses, lack of quantization of bone thickness to visualize ICA and impossibility to see conventional images and images under ICG-mode simultaneously.

Concerning the ventricular neuroendoscopic surgery field, the first experience was reported in 2013 by Wachter et al. [23]. The study included 11 patients with non-communicating hydrocephalus undergoing endoscopic third ventriculostomy (ETV). The authors used a prototype of a video-integrated 0° rigid endoscope with a diameter of 5.8 mm that replaced a standard ventriculoscope only in the step of the opening of the third ventricle floor. In this step, a dose of 0.2–0.5 mg/kg of ICG was injected. No adverse events related to ICG administration occurred. Except a case of technical failure, e-ICG facilitated the identification of the basilar tip and its branches especially in the presence of an opaque third ventricular floor (*n* = 5).

Tsuzuki et al. [22] reported, in 2013, three cases of hydrocephalus secondary to intra- and periventricular tumors. All patients underwent endoscopic transventricular biopsy and ETV. The authors used an ICG telescope 5.8 mm/19 cm in addition to a flexible endoscope. The ICG dose was 12.5 mg, administered in single boli twice for two cases and single boli of 7.5 + 12.5 mg in the latter cases. There were no side effects related to ICG administration. E-ICG helped to identify the tumor margins and to detect the differences of intratumoral ICG accumulation so to consider an adequate region for tumor biopsy.

In the neurovascular field, the first application of e-ICG was reported in 2012 by Bruneau M et al. [16]. This technique was applied during a case of unruptured anterior communicating artery aneurysm for which the patient was scheduled to undergo a conventional microsurgical clipping procedure. The endoscopic used was a 0° (19 cm, 5.8 mm) NIR-optimized endoscope, with a built-in optical filter. Interestingly, the integrated filter did not suppress the microscopic illumination light which inhibited the microscopic and endoscopic ICG-mode to be used in tandem. A 20-mg bolus of ICG was administered twice following microscopic assisted dissection, and two boli were subsequently administered after clipping (for microscopic and endoscopic ICG-mode, respectively). E-ICG offered high-resolution, panoramic view, and consequently, additional information about aneurysm occlusion and patency of parent and perforating vessels.

The most relevant paper concerning the vascular group was reported by Mielke et al. [17] in 2014. The same group pioneered the use of e-ICG in ventricular neuroendoscopic surgery field [23]. This study represents to date the largest sample series including 26 patients with unruptured and ruptured cerebral aneurysms in which the authors compared the information given by m-ICG and e-ICG. They used a prototype of a video-integrated 0° rigid endoscope with a 5.8-mm diameter. ICG was administered before and after clip placement with the dose of 0.2–0.5 mg/kg. No adverse events and technical failures related to e-ICG occurred. Compared with m-ICG, e-ICG provided more information in 11 of the 26 operations (42.3%), mostly due to longer duration of fluorescence (10 times longer), different viewing angles, and closer enlarged view of the vessels and aneurysm.

Yoshioka et al. [27] reported in 2015 nine cases of cerebral aneurysms underwent microsurgical clipping with e-ICG assistance. This series includes three patients already considered by the same group in a paper reported in 2012 by Nishiyama Y et al. [18]. The authors used 30°/70° rigid endoscopes with 4-mm-diameter and a cutoff filter to visualize fluorescence. ICG dose was not reported; however, the given dose was administered pre- and post-clipping. No complications related to additional use of e-ICG were reported. In one case, an adequate endoscope position during surgery was difficult due to limited space. E-ICG appeared helpful to evaluate the blood flow in perforating arteries behind parent arteries or aneurysm itself.

In the brain tumor field, e-ICG applications were restricted to a single case only. Yokoyama et al. [26] in 2016 reported their experience with e-ICG for superselective intra-arterial chemotherapy in recurrent skull base tumors. The authors used a 0° endoscope (5.8 mm/19 cm) and a dose of 5 mg of ICG. They performed an angiography after identification of a branch of a possible tumor feeding artery. In this phase, e-ICG was additionally used for confirmation of arteries supplying tumors and to decide about the administration of anticancer drug. Among visualization supported modules, SPECTRA-A mode revealed the blood supply to tumors more accurately than standard mode. Interestingly, the authors observed an ICG penetration within the tissue up to 10 mm.

## Discussion

During the last years, indocyanine green angiography has become a convincing technology in several neurosurgical fields to limit the morbidity and improve clinical outcomes [28–32]. In our experience, the enhanced vascular anatomical visualization during various endoscopic procedures was useful in all clinical cases presented in the study to visualize arterial, capillary and venous structures that can be precisely observed intraoperatively in real time. The use of such tool demonstrated great versatility in different surgical scenarios and complex vascular environments by the main advantage of endoscopy: enhanced visualization, better magnification of the areas of interest and dynamic vision “around the corner”.

As a matter of fact, the results of our systematic literature review and our institutional experience allowed to highlight few points.

The main factor is represented by a prolonged ICG fluorescence visualization compared to the traditional one visualized via the fluorescence mode of the operating microscope. Such unique features open new and extraordinary applications beyond the use of m-ICG in different surgical fields due to the longer duration of fluorescence, 10 times more [17] and up to 35 ± 7 min in our experience using a single ICG administration, i.e., during pituitary adenomas surgery. Indeed, the pituitary gland remained fluorescent until the end of the procedure so to safely preserve it during tumor removal [20]. Furthermore, the ICG depth of light energy showed the singular and extraordinary possibility of penetration within the tissues, thus identifying vascular structures masked under different anatomical layers up to 3–10 mm below the tissues’ surface [26, 33]. Using such intrinsic propriety during endoscopic procedures, it was possible to enhance different vascular structures depending on the surgical area of interest: the nasal mucosa to localize the posterior nasoseptal branch of the sphenopalatine artery in order to raise vascularized nasal flaps during extended endoscopic endonasal approaches; the bone to visualize the parasellar and paraclival segments of the internal carotid artery and the dura mater to localize the superior and inferior intercavernous sinuses during endoscopic endonasal approaches; and the other application in neuroendoscopy allowed to see beyond the third ventricular floor in order to identify the position of the basilar and posterior cerebral arteries before performing a third ventriculostomy [23] (Video 1).

The other significant aspect of our pilot study is to report our experience on the new alternative endoscopic technique applications of ICG apart from the vascular field, such as endoscopic endonasal, ventricular and brain tumor surgeries. The idea was to demonstrate the versatility of such tool in multiple surgical scenarios in which the use of e-ICG may complement other intraoperative enhanced visualization techniques related to regional blood flow and capillary density assessment relative to variations in tissue metabolic activity (i.e., fluorescence-guided surgery with 5-aminolevulinic acid or fluorescein) [20]. In such new fields, the use of e-ICG will open new surgical strategies for skull base reconstruction techniques, i.e., the harvesting of vascularized nasoseptal flaps tailoring the inclusion of the septal branch of the sphenopalatine artery. Indeed, the powerful possibility of e-ICG to enhance the “individual anatomy” of vascular structures in real time during the surgical procedures represents an important complement to help achieve the highest levels of safety while minimizing neurologic injury. In this regard, the visualization of the basilar and posterior cerebral arteries during third ventricular surgery is the key point to determine the correct site for ventriculostomy ensuring total safety.

Another aspect is the possibility to detect blood-brain barrier disruption. Employing the e-ICG during intrinsic brain tumor surgery located within the cortical surface enabled the identification of the tumor based on the differences in fluorescence intensity, while in extraaxial tumors, the e-ICG open the possibility to localize peritumor arteries and to the preserve normal anatomy.

Despite the many viable and extraordinary features of e-ICG, such tool requires further development. A smaller diameter endoscope may be more useful to a relative 5.8 mm in diameter telescope used in the present study which could improve surgical dexterity and the surgical freedom [34] especially during endoscopic endonasal and ventricular surgery in which the operative field is extremely confined. Another limitation of the endoscope is the unavailability of variable angled scopes to enable direct operative view of blind corners. One more significant limitation is the absence of a dedicated ICG-integrated endoscope with a working channel and adequate diameter for ventricular procedures. We note that a standard ventriculoscope, coupled with an “external” ICG optical filter, does not provide the same image quality exhibited in procedures using an integrated ICG endoscope. Another critical point is the need to obtain a very clear surgical field especially during endoscopic endonasal surgery in order to expose the desired surgical region without the risk of false visual perception due to the bleeding/oozing that can create “false” vascular structures. Indeed during e-ICG visualization, the constant use of suction over the area of interest is the key maneuver to distinguish an artery rather than an extravascular fluorescein blood clot mimicking a vascular structure. In such situations, the possibility to have a simultaneous real-time visualization of fluorescent and non-fluorescent images on screen would have been very useful but is lacking in the current e-ICG technology available. General aspects and pros and cons of e-ICG use are summarized in Table 2.

Finally, the usefulness of e-ICG angiography in different fields of neurosurgery is increasingly acknowledged and there is still room for technical developments as more applications are developed and more experience is gained.

## Conclusions

E-ICG presents as a safe and useful method, providing real-time and prolonged vascular information that can contribute to a significant improvement to clinical outcomes and consequently a reduction of major risks in several fields of neurosurgery. Future advancements of the e-ICG technology will improve the potential for the technique to become a critical tool for different applications in neurosurgery.

## Electronic supplementary material

Video 1 Intraoperative video demonstrating multimodal applications of e-ICG during different endonasal, intraventricular, aneurysm and brain tumor surgeries. (MOV 38166 kb)
